# DeepACLSTM: deep asymmetric convolutional long short-term memory neural models for protein secondary structure prediction

**DOI:** 10.1186/s12859-019-2940-0

**Published:** 2019-06-17

**Authors:** Yanbu Guo, Weihua Li, Bingyi Wang, Huiqing Liu, Dongming Zhou

**Affiliations:** 1 School of Information Science and Engineering, Yunnan University, Kunming, 650091 China; 2 Research Institute of Resource Insects, Chinese Academy of Forestry, Kunming, 650224 China

**Keywords:** Protein secondary structure, Deep learning, Asymmetric convolutional neural network, Long short-term memory

## Abstract

**Background:**

Protein secondary structure (PSS) is critical to further predict the tertiary structure, understand protein function and design drugs. However, experimental techniques of PSS are time consuming and expensive, and thus it’s very urgent to develop efficient computational approaches for predicting PSS based on sequence information alone. Moreover, the feature matrix of a protein contains two dimensions: the amino-acid residue dimension and the feature vector dimension. Existing deep learning based methods have achieved remarkable performances of PSS prediction, but the methods often utilize the features from the amino-acid dimension. Thus, there is still room to improve computational methods of PSS prediction.

**Results:**

We propose a novel deep neural network method, called DeepACLSTM, to predict 8-category PSS from protein sequence features and profile features. Our method efficiently applies asymmetric convolutional neural networks (ACNNs) combined with bidirectional long short-term memory (BLSTM) neural networks to predict PSS, leveraging the feature vector dimension of the protein feature matrix. In DeepACLSTM, the ACNNs extract the complex local contexts of amino-acids; the BLSTM neural networks capture the long-distance interdependencies between amino-acids. Furthermore, the prediction module predicts the category of each amino-acid residue based on both local contexts and long-distance interdependencies. To evaluate performances of DeepACLSTM, we conduct experiments on three publicly available datasets: CB513, CASP10 and CASP12. Results indicate that the performance of our method is superior to the state-of-the-art baselines on three publicly datasets.

**Conclusions:**

Experiments demonstrate that DeepACLSTM is an efficient predication method for predicting 8-category PSS and has the ability to extract more complex sequence-structure relationships between amino-acid residues. Moreover, experiments also indicate the feature vector dimension contains the useful information for improving PSS prediction.

## Background

Protein secondary structure (PSS) is the 3-dimensional form of local segments in protein sequences [1, 2], and secondary structure elements unaffectedly form as an intermediate before the protein sequence folds into its tertiary structure. The prediction of PSS is a vital intermediate step in tertiary structure prediction and is also regarded as the bridge between the protein sequence and tertiary structure [3, 4]. The accurate identification of PSS cannot only enable us to understand the complex dependency relationships between protein sequences and tertiary structures, and also promote the analysis of protein function and drug design [3, 5–7]. The experimental identification of PSS is expensive and time consuming, and thus it becomes urgent to develop efficient computational approaches for predicting PSS based on sequence information alone. However, accurately predicting PSS from sequence information and understanding dependency relationships between sequences and structures are a very challenging task in computational biology [3, 4, 8].

PSS often is classified into 3 categories: H (helices), E (strands) and C (coils); in addition, according to the DSSP program [9], PSS is also classified into 8 categories: G (3-turn helix), H (4-turn helix), I (5-turn helix), T (hydrogen bonded turn), E (extended strand in parallel and/or anti-parallel β-sheet conformation), B (residue in isolated β-bridge), S (bend) and C (coil). Of course, the methods of PSS prediction [3, 4] are also commonly classified into 3-category prediction and 8-category prediction. Compared to 3-category prediction, the prediction of 8-category secondary structure can reveal more detail structure information of proteins and the task is also more complex and challenging. Thus, this paper only focuses on 8-category PSS prediction based on protein sequences.

PSS prediction has been extensively studied [6]. Many computational methods have also been proposed to identify secondary structures, such as statistical methods [10], SVM [11], CRF [12], and the methods have achieved remarkable performances. Statistical methods [10] were used to identify the secondary structures by analyzing the probability of the specific amino acid, but their performances are far from the application due to the inadequate features extracted. Subsequently researchers [11, 13] also proposed secondary structure prediction methods based on SVM or SVM variation. Although the methods have been used successfully, both statistical models and traditional machine learning methods have their own limitations. In brief, traditional methods heavily rely on handcrafted features and easily ignore the long-distance dependencies of protein sequences.

Inspired by the remarkable success in computer vision [14], speech recognition [15] and sentiment classification [16], deep learning based methods are now being intensively used in many biological research fields, such as protein contact map [17], drug-target binding affinity [18, 19], chromatin accessibility [20] and protein function [21, 22]. The main advantages of deep learning methods are that they can automatically represent the raw sequence and learn the hidden patterns by non-linear transformations. Moreover, these convolutional neural networks (CNNs) and recurrent neural networks (RNNs) models have already been applied to the PSS prediction [3, 4, 8, 23, 24].

It’s well known that the dependencies between amino-acid residues usually contain local contexts and long-distance interdependencies [3, 4, 24] in protein sequences. Consequently, according to the dependencies between amino-acid residues, deep learning based methods can be classified into three categories: local context based methods, long-distance dependency based methods, local context and long-distance dependency based methods. Firstly, local context based methods indicated that the methods usually identified the secondary structure of each amino acid based on the local contexts or statistical features in protein sequences. Pollastri et al. [25] proposed a prediction method, called SSpro8, based on PSI-BLAST-derived profiles by bidirectional recurrent neural networks (BRNNs). Wang et al. proposed a conditional neural field (CNF) prediction method. Secondly, long-distance dependency based methods indicated that the methods mainly focused on the long-distance dependency of between amino-acid residues. Sønderby et al. [26] utilized bidirectional long short-term memory (BLSTM) to capture the long-distance dependency of between amino-acid residues for PSS prediction. Finally, local context and long-distance dependency based methods indicated that the methods exploited both local contexts and long-distance dependencies to predict PSS. Zhou et al. [6] presented a new supervised generative stochastic network (GSN) prediction method. Guo et al. presented a hybrid deep learning framework integrating two-dimensional CNNs with bidirectional recurrent neural networks. Zhou et al. [8] proposed an end-to-end deep network method, which was called a deep convolutional and recurrent neural network (DCRNN) leveraging cascaded convolutional and recurrent neural networks. Zhang et al. [4] presented a novel deep learning architecture, called convolutional residual recurrent neural networks (CRRNNs), leveraging convolutional neural networks, residual networks, and bidirectional recurrent neural networks. Zhou et al. [3] presented a novel deep learning model, called CNNH, by utilizing multiple CNNs with the highway network.

Compared to traditional machine learning methods, deep learning based methods can automatically extract amino acid features and hidden patterns in protein sequences. The feature representation of each amino-acid sequence usually forms the matrix, and it’s obvious that the matrix contains two dimensions (rows correspond to amino acid dimensions, and columns correspond to feature vector dimensions). CNNs based secondary structure prediction methods [3, 4] have achieved remarkable results. However, the methods only capture features along the amino-acid residue dimension. Thus, the methods may ignore some important features, which are hidden in the feature vector dimension of protein sequences and likely to be useful for predicting the secondary structures.

Inspired by the success of asymmetric convolutional neural networks (ACNNs) [27] and ultra-deep neural networks [17] in protein contact map prediction, we propose a novel method, called DeepACLSTM, to predict 8-category PSS. DeepACLSTM efficiently applies ACNNs combined with BLSTM neural networks to predict PSS, leveraging the feature vector dimension of the protein feature matrix. The main contributions of this work include: (1) the asymmetric convolutional operation is used to extract complex local contexts between amino-acid residues in protein sequences. Moreover, two stacked BLSTM neural networks are used for further extracting the long-distance interdependencies between amino-acid residues. (2) To verify the efficacy of our DeepACLSTM, we carry out 8-category PSS prediction experiments on three public test datasets respectively: CB513, CASP10 and CASP11. Experiments demonstrate that our proposed method consistently outperforms other benchmark methods. In addition, experiments also indicate that the feature vector dimension contains the useful information for improving 8-category PSS prediction.

## Results

### Overview of DeepACLSTM

**Fig. 1:**
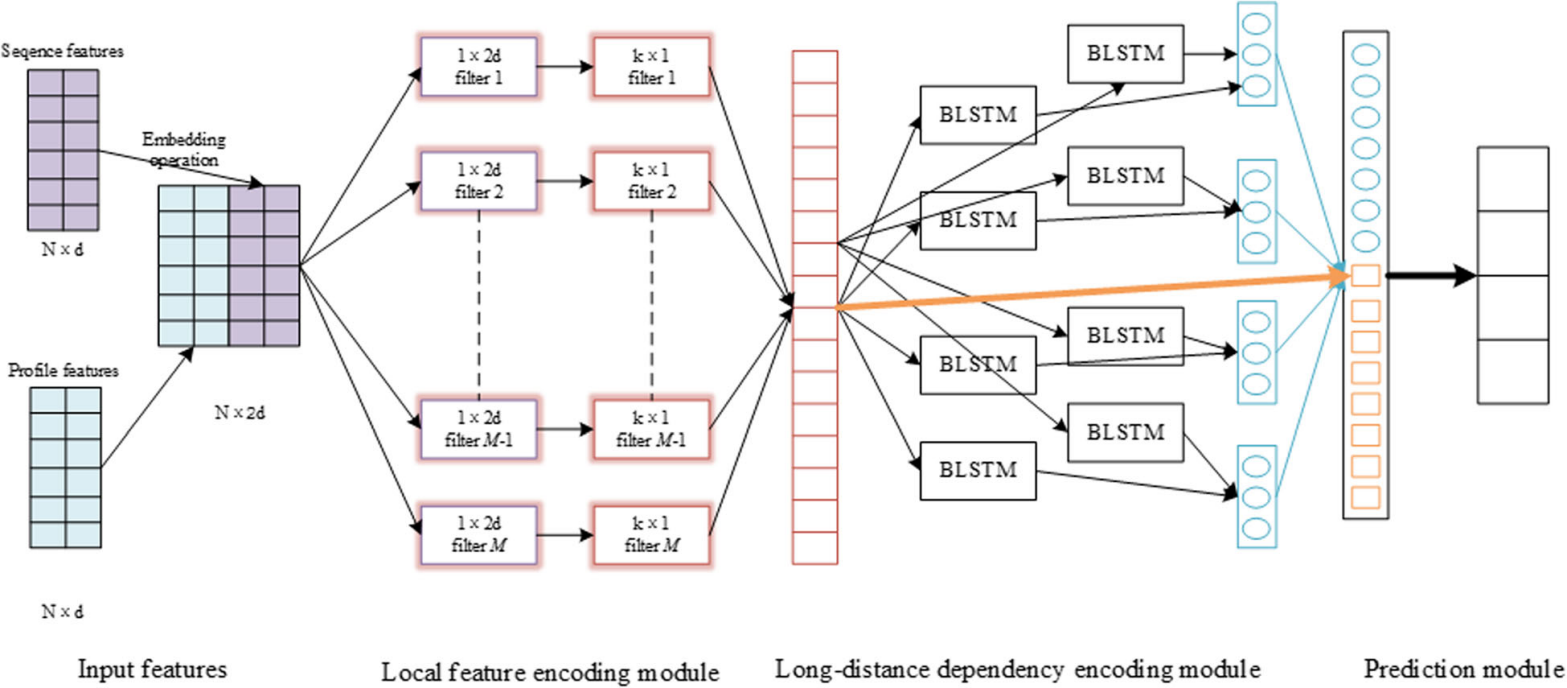
Overview of DeepACLSTM structure

As illustrated in Fig. 1, our proposed deep asymmetric convolutional long short-term memory neural model, called DeepACLSTM, comprises of three modules: Local feature encoding module, Long-distance encoding module and Prediction module.

In DeepACLSTM, sequence features and profile features are first concatenated into the matrix representation of proteins. The local feature encoding module maps the matrix into the local dependency feature of amino-acid residues by asymmetric convolution filters that include two convolutional filters: 1 × 2*d* convolutional filters and *k* × 1 convolutional filters. Asymmetric convolutional filters first scan along the input for capturing the low level feature patterns of protein sequences by 1 × 2*d* convolutional operations with *M* filters; and then subsequent *k* × 1 convolutional operations with *M* filters further project the low level feature patterns from 1 × 2*d* convolutional filters to high level local dependency patterns by *k* × 1 convolutional filters. The long-distance dependency encoding module captures long-distance dependencies from the representation extracted by the local feature encoding module using two stacked BLSTM neural networks.

The prediction module takes the representation generated by the local feature encoding module and the long-distance dependency encoding module as input, and then predicts 8-category secondary structure of each amino-acid residue through the softmax function. In our model, the fully connected layer with a rectified linear unit (ReLU) reduces input features to a low dimension, for the purpose of alleviating computational burden and meanwhile facilitating the extraction of high level features. Moreover, input features are also discarded at random by the dropout operation [28].

### Implementation of DeepACLSTM

A distinguishing characteristic of our model is the use of asymmetric convolutional operations and BLSTM. Asymmetric convolution operations contain two types of filters, as showed in Fig. 1. Benefitting from the rapid development of deep learning toolbox, we can easily use the high level neural network API tool (Keras, https://github.com/fchollet/keras) to design an abstract model, and the backend of Keras is Tensorflow.

Firstly, we develop our proposed DeepACLSTM by Keras API. For example, 1 × 2*d* convolutional filters are implemented by the Convolution1D layer and *k* × 1 convolutional filters are implemented by the convolution2D layer from Keras. The stacked BLSTM is implemented by the LSTM layer from Keras.

Secondly, we train the model and update the parameters in DeepACLSTM using the adaptive moment estimation (Adam) algorithm [29]. The datasets and the codes of our method can be accessed online at https://github.com/GYBTA/DALSTM/. Finally, Table 1 shows our proposed deep learning based methods typically have various parameters. In Table 1, FC represents the fully connected layer and NP represents the number of parameters.

**Table 1:** The main structures and parameters of DeepACLSTM

Layer Type	Size	NP
embedding	21	441
Convolution1D	1 × 42	1806
Convolution2D	3 × 1	168
Dropout1	0.5	0
*FC* ^1^	400	706,000
${LSTM}_f^1$	300	841,200
${LSTM}_b^1$	300	841,200
${LSTM}_f^2$	300	721,200
${LSTM}_b^2$	300	721,200
Dropout2	0.4	0
*FC* ^2^	600	600,600
Softmax	8	4808

### Evaluation metrics

The Q8 accuracy is the main evaluation metric in 8-category secondary structure prediction [3, 8]. This paper only focuses on 8-category PSS prediction, so the performance of our model is also evaluated by Q8 accuracy, which is the percentage of the amino-acid residues predicted correctly. A bigger value indicates a better performance of PSS prediction.

### Experimental settings

As shown in Fig. 1, the input of DeepACLSTM is a *N* × *d* matrix, where *N* is the length of the input sequence and *d* is the dimension of vectors. In our work, in order to deal with sequences and compare performance with other baseline methods conveniently [3, 6], all the protein sequences are normalized to *N* (*N* = 700) amino acids in the training, validation and test dataset. In other words, for all the datasets, protein sequences shorter than 700 amino acids are padded with zero vectors. Sequences longer than 700 amino acids are truncated for the training and validation dataset. For protein sequences longer than 700 amino acids in the test dataset, we split them to two overlapping sequences.

To prevent our method from overfitting, *L*2 regularization, Dropout [28] techniques and early-stopping methods are exploited during training our DeepACLSTM. The dropout is first applied between the local feature encoding module and the long-distance dependency module. Then the dropout is applied between the prediction module and the long-distance dependency module. We also adopt the early-stopping method with the maximum number of iterations, and it would stop training the model after 5 times of the unimproved loss value on the validation set. The DeepACLSTM is trained on a single NVIDIA GeForce GTX 1060 GPU with 6GB.

### The choice of input features

In the section, we analyze whether both the sequence features and profile features are necessary to predict PSS. Thus, we conduct three experiments on CB513 dataset. The parameters of DeepACLSTM are shown in Table 1. The first experiment evaluates DeepACLSTM with sequence features and the Q8 accuracy is 57.1%; the second experiment evaluates DeepACLSTM with profile features and the Q8 accuracy is 69.6%; moreover, the third experiment evaluates DeepACLSTM with sequence and profile features and the Q8 accuracy is 70.5%.

**Fig. 2:**
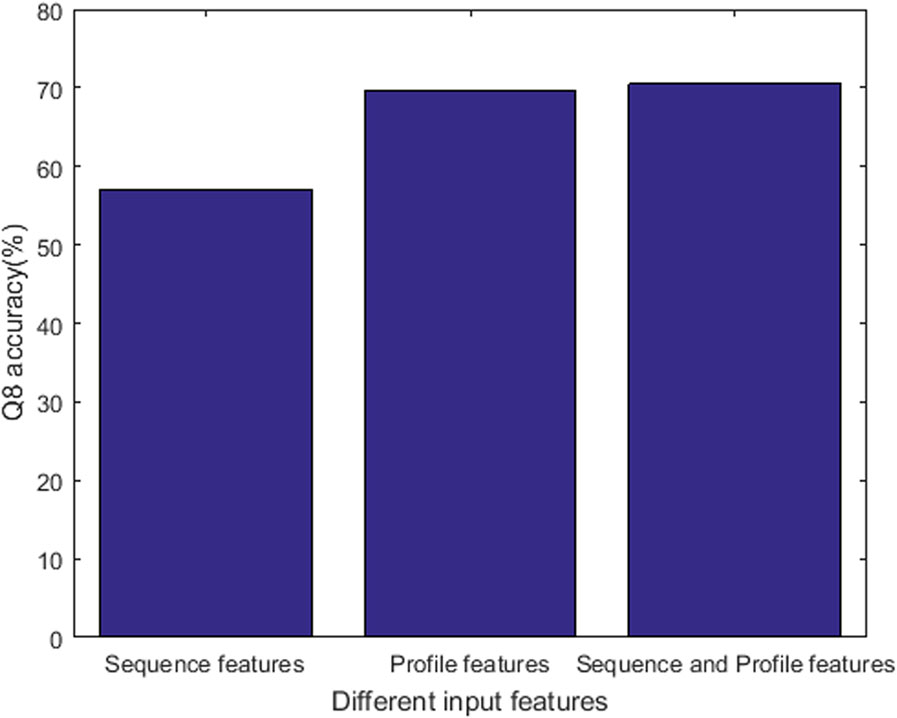
The performance of DeepACLSTM on different input features

The results in Fig. 2 show that DeepACLSTM can obtain the best performance when both sequence and profile features are used as the input features. Thus, we regard sequence and profile features as the input features of our method.

### Results of DeepACLSTM

We mainly exploit four protein datasets, which consist one training dataset called CB5534 and three publicly available test datasets: CB513, CASP10 and CASP11. Their details are described in section “Methods”. For validation datasets, we randomly divide CB5534 into the training set and the validation set. We train our model on the CB5534 and compare the Q8 accuracy of our method with the baseline methods on three public test datasets: CB513, CASP10 and CASP11.

Experimental results of DeepACLSTM are summarized in Table 2 and Table 3 on the test datasets in detail. Table 2 shows the performance of DeepACLSTM with different LSTM output dimensions ranging from 50 to 500. Table 3 shows the performance of DeepACLSTM with different filter sizes from 3 to 21. From Table 2, we can see that our method obtains the best Q8 accuracy when the output dimension of LSTM is 300. When the output dimension of LSTM is increased to 300, the Q8 accuracy is increased obviously, and then the accuracy starts to decrease. The main reason may be that our method could capture the most long-distance dependency information when the output dimension is increased to 300 in LSTM. While the output dimension of LSTM is bigger or smaller than 300, our method cannot capture more information of residues in protein sequences. Thus, the LSTM output dimension of our method is 300 in our model.

**Table 2:** The Q8 accuracy (%) of DeepACLSTM with different LSTM units and the best values are marked in bold

LSTM output dimension	CASP10	CASP11
50	71.8	70.2
100	72.4	70.5
150	74.2	72.1
200	74.1	72.2
250	74.5	72.3
300	**75.0**	**73.0**
350	73.7	71.8
400	73.8	71.6
450	74.8	71.8
500	72.1	70.1

**Table 3:** The Q8 accuracy (%) of DeepACLSTM with different filter size and the best values are marked in bold

Filter Size	CASP10	CASP11
3	**75.0**	**73.0**
5	73.9	72.1
7	74.2	72.1
9	74.7	72.4
11	74.4	72.3
13	71.3	70.0
15	69.6	68.6
17	74.3	72.3
19	73.5	71.6
21	74.0	71.7

From Table 3, we can find that our method can get the best Q8 accuracy when the filter size is 3. The Q8 accuracy decreases gradually with the increase of the filter size. When the filter size is increased, the local feature encoding model can extract local correlations between more remote amino-acid residues, but the Q8 accuracy of DeepACLSTM is decreased. The reason is possible that the bigger convolutional filter size integrated with BLSTM neural networks cannot extract more amino-acid features. Thus, the filter size of the local feature encoding module is 3 in our model.

### Comparison with baseline methods

PSS is critical for analyzing protein function and drug design [3, 30]. Many computational methods have been proposed for improving the performance of PSS prediction. In this paper, we compare our method with the following approaches:

**† SSpro8**: Pollastri et al. [25] used ensembles of bidirectional recurrent neural network architectures and PSI-BLAST-derived profiles to improve the prediction of 8-category PSS.

**† CNF**: Wang et al. presented a new probabilistic method for 8-category secondary structure prediction using a conditional neural field (CNF). The CNF prediction method not only models the complex relationship between sequence features and secondary structures, but also exploits the interdependencies among secondary structure types of adjacent residues.

**† DeepCNF**: Wang et al. [31] proposed an extension method of CNF (DeepCNF) based on deep learning techniques, which was an integration method between CNF and shallow convolutional neural networks. DeepCNF could extract both complex sequence structure relationships and interdependencies between adjacent secondary structures.

**† GSN**: Zhou et al. [6] presented a new supervised generative stochastic network (GSN) based method to predict local secondary structure with deep hierarchical representation, which learned a Markov chain to sample from a conditional distribution.

**† DCRNN**: Li et al. [8] proposed an end-to-end deep network that predicted 8-category PSS from integrated local and global features between amino-acid residues. The deep architecture utilized CNNs with different filter sizes to capture multi-scale local features and three staked gate recurrent units to capture global contextual features.

**† CNNH**: Zhou et al. [3] presented a novel deep learning based prediction method for PSS, called CNNH, by using multi-scale CNNs with the highway network. Their deep architecture has a highway between two neighbor convolutional layers to deliver information from the current layer to next layer to capture contexts between amino-acid residues.

**† CBRNN:** Guo et al. [32] presented a hybrid deep learning framework integrating two-dimensional CNNs with bidirectional recurrent neural networks for improving the accuracy of 8-category secondary structure prediction.

In Table 4, the Q8 accuracies of SSpro8, CNF and DeepCNF are reported by Wang et al. [23] (2016) and Guo et al. reported the Q8 accuracy of CBRNN [32] (2018).

**Table 4:** The Q8 accuracy (%) of our method and baseline methods and the best performance are marked in bold

Methods	CB513	CASP10	CASP11
SSpro8	63.5	64.9	65.6
CNF	64.9	64.8	65.1
DeepCNF	68.3	71.8	72.3
CBRNN	70.2	74.5	72.5
DeepACLSTM	**70.5**	**75.0**	**73.0**

We first compare our method with the SSpro8, CNF, and DeepCNF. The methods mainly extract local contexts between amino-acid residues. Their results are shown in Table 4. From the Table 4, we can see that the Q8 accuracy of our method obviously outperforms the baseline methods on three public datasets; moreover, we can also find that the Q8 accuracy of DeepACLSTM increases by 2.2, 3.2 and 0.7% respectively than DeepCNF on CB513, CASP10 and CASP11 datasets. The outperformance indicates that DeepACLSTM can extract more long-distance interdependencies for improving the performance of 8-category secondary structure prediction. Compared to CBRNN, the performance of DeepACLSTM increases by 0.3, 0.5 and 0.5% on CB513, CASP10 and CASP11 respectively, which indicates that more local structural information can be captured by the asymmetric convolution.

In addition, we also compare DeepACLSTM to the baseline methods on CB513 and CB6133 datasets, including GSN, DCRNN and CNNH. The baseline methods cannot only extract the local contexts, and also capture long-distance dependency in protein sequences. Their results are shown in Table 5. From Table 5, the Q8 accuracy of our method increases by 0.2 and 0.2% than CNNH on CB513 and CB6133 datasets respectively. The outperformance indicates that asymmetric convolution can extract more local contexts between amino-acid residues and BLSTM neural networks integrated with asymmetric convolutions can extract more long-distance dependency information than CNNs with the highway.

**Table 5:** The Q8 accuracy (%) of our method and baseline methods and the best performance are marked in bold

Methods	CB6133	CB513
GSN	72.1	66.4
DCRNN	73.2	69.4
CNNH	74.0	70.3
DeepACLSTM	**74.2**	**70.5**

In Table 5, the Q8 accuracy of GSN is reported by Zhou et al. [6] (2014), the Q8 accuracy of DCRNN is reported by Li et al. [8] (2016) and the Q8 accuracy of CNNH is reported by Zhou et al. [3] (2018).

### Influence of the dropout settings

In the section, we explore that how different dropout rates and dropout settings impact on learning robust and effective features in protein sequences. Specially, our model contains two types of dropout settings: **dropout1** (D1) and **dropout2** (D2).

**Fig. 3:**
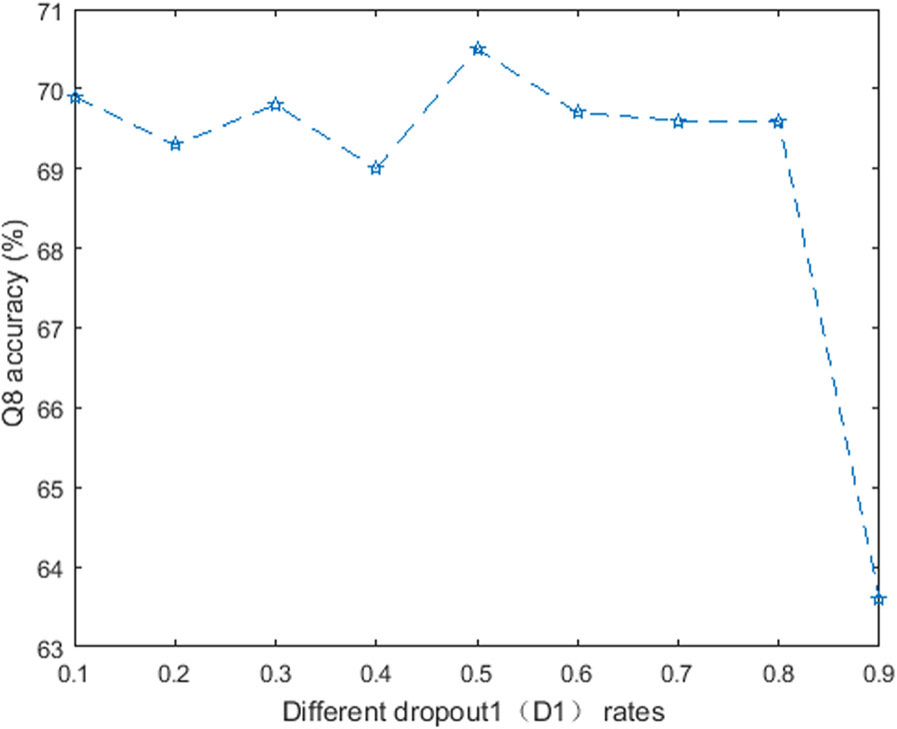
The performance of DeepACLSTM with different D1 rates

**Fig. 4:**
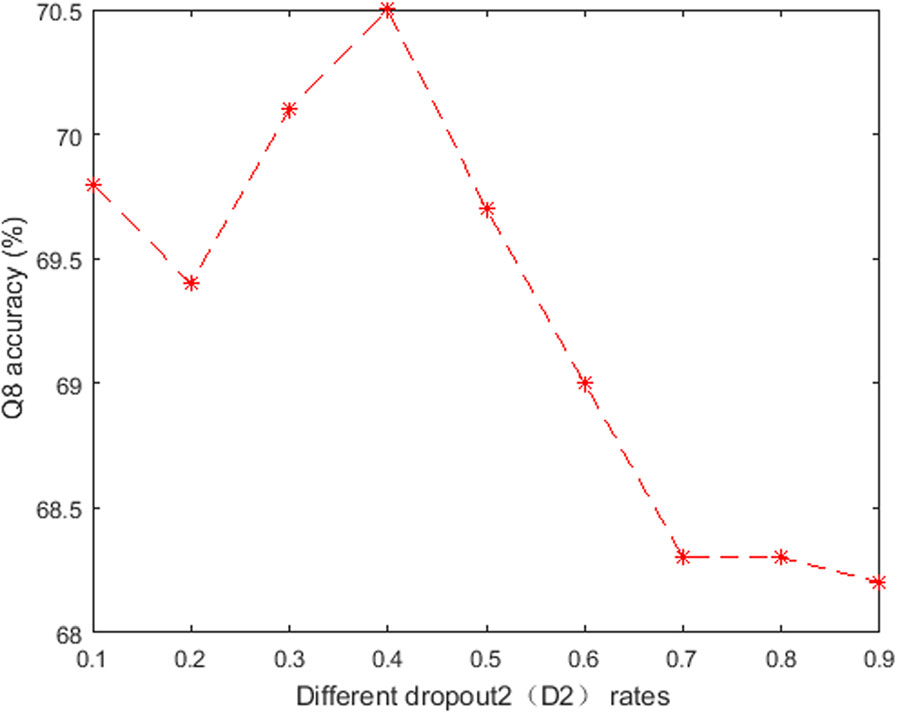
The performance of DeepACLSTM with different D2 rates

In order to obtain the optimal dropout rate, we first conduct two sets of experiments on CB513 based on the parameter settings in Table 1; and each dropout rate refers to a variable ranging from 0.1 to 0.9. Experimental results on CB513 dataset are listed in Fig. 3 and Fig. 4.

From Fig. 3, we can see that DeepACLSTM with the **D1** rate (*P* = 0.5) obtains the best Q8 accuracy. When the dropout rate *P* is bigger than 0.5, then the Q8 accuracy is decreased obviously. The main reason is possible that DeepACLSTM with the D1 rate (*P* = 0.5) can learn more robust and effective features between the local feature encoding module and the long-distance dependency module.

From Fig. 4, it’s obvious that DeepACLSTM with the D2 rate (*P* = 0.4) obtains the best Q8 accuracy between the prediction module and the long-distance dependency module. When the dropout rate is bigger than 0.4, then the Q8 accuracy is decreased obviously. The main reason is possible that DeepACLSTM with our model with D2 rate (P = 0.4) can learn more robust and effective features on the protein feature matrix.

Thus the D1 rate and the D2 rate are 0.5 and 0.4 in DeepACLSTM respectively. Moreover, in order to explore the influence of the dropout settings on DeepACLSTM with the parameter settings in Table 1, we conduct four experiments to get the appropriate dropout setting on test dataset CB513, CASP10 and CASP11. The four settings are YD1-YD2, YD1-ND2, ND1-YD2 and ND1-ND2, respectively. YD indicates the model adopts the dropout and ND indicates the model doesn’t adopt the dropout. Specially, YD1-YD2 shows that our method uses D1 and D2. YD1-ND2 shows that our method uses D1 and doesn’t use D2. ND1-YD2 shows that our method doesn’t use D1 and only uses D2. ND1-ND2 shows that our method doesn’t use D1 and D2.

The experimental results are shown in Table 6. As shown in Table 6, DeepACLSTM with YD1-YD2 achieves the best performance 70.5, 75.0 and 73.0% respectively on CB513, CASP10 and CASP11 dataset. From Table 6, we can see that the Q8 accuracy of our method with YD1-YD2 outperforms other settings on three public test datasets. Thus, we adopt the dropout setting to avoid overfitting and achieve the best performance in DeepACLSTM.

**Table 6:** The Q8 accuracy (%) of our method on different dropout settings

Dropout Setting	CB513	CASP10	CASP11
YD1-YD2	**70.5**	**75.0**	**73.0**
YD1-ND2	68.5	72.3	70.3
ND1-YD2	69.1	73.3	71.1
ND1-ND2	69.2	73.7	71.0

## Discussion

Compared to the baseline methods, DeepACLSTM utilizes ACNNs to learn the local contexts from the protein feature matrix during training the model. As shown in Fig. 1, the protein feature matrix is first delivered to the local feature encoding module, which is an asymmetric convolution containing 1-dimensional and 2-dimensional convolutional filters. The convolutional filters with 1 × 2*d* extract information from the feature vector dimension on each amnion-acid residue; and then features from convolutional filters with 1 × 2*d* are fed into the convolutional filters with *k* × 1 hat capture the adjacent *k* amino-acid residues of each position in protein sequences. As shown in Table 3, we also conduct 10 experiments of DeepACLSTM with different filter sizes ranging from 3 to 21 and it’s obvious that DeepACLSTM can achieve the best performance when the filter size is 3 in asymmetric convolutional operations. That’s to say, the asymmetric convolutional operation with adjacent 3 amino-acid residues can extract more local complex features in protein sequences. Secondly, the output of the local feature encoding module is organized as the local feature of protein sequences and then is fed into the long-distance dependency encoding module, which contains two stacked BLSTM neural networks. As shown in Table 2, we conduct 10 experiments of DeepACLSTM with different LSTM output dimension ranging from 50 to 500 and find DeepACLSTM can achieve the best performance when the LSTM output dimension is 300. In other words, the long-distance dependency encoding module with 300 LSTM output dimension has ability to learn more long-distance dependency based on the local features captured by the local feature encoding module.

Based on the above discussion, we can find that DeepACLSTM with different convolutional filter sizes and LSTM output dimensions can get different performances of predicting PSS based on sequence information, and the appropriate parameter adjustment can further improve the performance of the model.

## Conclusion

Understanding the complex dependency relations is a very important task in computational biology between sequences and structures. In order to predict 8-category PSS accurately, we have proposed a novel deep learning method for predicting PSS based on sequence information, called DeepACLSTM. Compared to the state-of-art methods, the performance of our method is superior to their performances on three public test datasets: CB513, CASP10 and CASP11. Experiments demonstrate that DeepACLSTM is an efficient method for predicting 8-category secondary structure. Moreover, experiments also indicate the feature vector dimension contains useful information for improving PSS prediction. Moreover, the asymmetric convolution integrated with BLSTM neural networks can extract more local contexts and more long-distance interdependencies between amino-acid residues in protein sequences, which are important to improve 8-category PSS prediction.

Residual neural networks achieved remarkable performance in PSS [4] prediction and protein contact map prediction [17]. Moreover, Zhang et al. [4] also utilized four types of input features, including a position-specific scoring matrix (PSSM), protein coding features, conservation scores, and physical properties, to characterize each residue in protein sequences. Inspired by Zhang et al. [4] and Wang et al. [17], in the future, we would improve our method from the following two aspects: (1) adding other additional properties in input features of proteins, such as physical properties, (2) extending the prediction model using residual networks.

## Methods

Firstly, we introduce four publicly available datasets that the models are trained and tested on. Then, we describe in detail the initial representation of amino-acid residues with the embedding technique, which aims to encode the discrete sequence feature into the continuous sequence feature. Moreover, we also describe asymmetric convolutional operations, containing two types of convolutional filters in detail, which is the components of the local context encoding module. The local context encoding module takes the amino-acid vector matrix as input and produces higher-level presentation of amino-acid residues in protein sequences; and then we introduce the stacked BLSTM neural networks which are used to incorporate local contexts on both sides of every amino-acid position to get the long-distance interdependencies in the input. Finally, two types of features are concatenated and fed into the prediction module.

## Data sources

We evaluate our method on three public test datasets: CB513, CASP10 and CASP11, which were previously used as the test datasets for PSS prediction [3, 4, 8]. The details of datasets are as follows.

### CB6133 dataset

The CB6133 [33] dataset was produced by PISCES CullPDB [34] and was a non-homologous dataset with known secondary structures. CB6133 contains 6128 protein sequences. When the dataset is used to test the model, 5600 proteins are regarded as the training dataset, and 256 proteins are regarded as the validation dataset and 272 proteins are regarded as the test dataset.

### CB513 dataset

The CB513 [33] dataset contains 514 protein sequences and is widely regarded as a test dataset [3, 8] for PSS prediction.

### CASP10 and CASP11 dataset

The CASP10 and CASP11 [3, 8] datasets contain 123 and 105 protein sequences, respectively. They are often regarded as the test datasets.

Since there exists some redundancy between CB6133 and CB513 datasets, the CB513 dataset cannot be used to evaluate the models directly. Therefore, sequences over 25% similarity need to be filtered in CB6133 between CB6133 and CB513; finally, the new dataset achieved is named as **CB5534 dataset** and it contains 5534 protein sequences. When the performance of DeepACLSTM is evaluated on test datasets: CB513, CASP10 and CASP11, 5278 proteins of the CB5534 are randomly chosen as the training dataset, and other proteins are regarded as the validation dataset, which aims at optimizing the parameters of the model during training the model.

### Input features

DeepACLSTM takes the feature sequence of a given protein as input, and predicts the corresponding secondary structure labels of amino acids. For each amino acid in a protein sequence, its input feature is a *2d* (*d* = 21) dimensional vector, which concatenates the sequence feature and profile feature [3, 8, 33]. As shown in Fig. 1, the sequence feature is a *d*-dimensional vector encoding the type of the amino acid in a protein, and the profile feature is also a *d*-dimensional vector, called the position special scoring matrix (PSSM). In DeepACLSTM, the profile feature was generated by the PSI-BLAST [35] and rescaled by a logistic function [36].

In addition, the sequence feature vector is a sparse one-hot vector, while the profile feature vector is a dense vector. In order to avoid the influence of feature inconsistency, we also transform the sparse sequence features to the dense sequence features by an embedding operation from Keras (https://github.com/fchollet/keras). As shown in Fig. 1, after the embedding operation and concatenating operation, we obtain the sequence features with size of *N* × 2*d*.

### Local feature encoding module

Convolutional neural networks (CNNs) often contain three convolutional operations: 1-dimensional convolutional operations, 2-dimensioanl convolutional operations and 3-dimensional convolutional operations. 1-dimensional convolutional operations are usually used for dealing with sequence data, such as sentiment analysis and sequence structure prediction [16, 23, 27]; Moreover 2-dimensional and 3-dimensional convolutional operations are often used to capture spatiotemporal feature in image recognition and video classification [37–39]. CNN based methods [3–5] have been applied in PSS prediction and achieve remarkable successes. Nevertheless, the methods often ignore features from the feature vector dimension, which may be useful for improving the performance of PSS prediction.

In our method, the local feature encoding module exploits the asymmetric convolution to extract the local hidden patterns and features of adjacent amino-acid residues from the input matrix. This module contains 1-dimesnional convolutional operations and 2-dimensional convolutional operations, as shown in Fig. 1.

Instead of exploiting *k* × 2*d* convolutional operations described in Kim [40], we factorize *k* × 2*d* convolution operations into 1 × 2*d* convolution operations followed by the *k* × 1 convolution operations, as utilized by Liang et al. [27] and Wang et al. [17].

Let *x*: *x*_1_*x*_2_*x*_3_⋯*x*_*N* − 2_*x*_*N* − 1_*x*_*N*_ denotes the protein sequence with *N* amino-acid residues. Generally, let *x*_*j* : *j* + *i*_ refer to the concatenation of amino acids *x*_*j*_, *x*_*j* + 1_, ⋯, *x*_*j* + *i* − 1_, *x*_*j* + *i*_. As shown in Fig. 1, the convolutional operation corresponding to the 1 × 2*d* convolutional operation with the filter *W*^1^ ∈ ℝ^1 × 2*d*^ is applied to each amino acid *x*_*j*_ in protein sequences and generates a corresponding feature ${c}_j^1$:

1 $${c}_j^1=f\left({W}^1\otimes {x}_j+{B}^1\right)$$

where ⊗ is element-wise multiplication, *B* is a bias term and *f* is a non-linear function such as the sigmoid, hyperbolic tangent and rectified linear unit. In this paper, we chooses rectified linear unit (ReLU) [41] as the nonlinear function; Then we can get the feature map *c*^*1*^:

2 $${c}^1=\left[{c}_1^1,{c}_2^1,{c}_3^1,\cdots, {c}_{N- 2}^1,{c}_{N- 1}^1,{c}_N^1\right]$$

As shown in Fig. 1, after the 1 × 2*d* convolution, the second convolutional operation corresponding to the *k* × 1 convolution with the filter *W*^2^ ∈ ℝ^*k* × 1^ is exploited to the window of *k* features in the feature map *c*^1^ to produce the new feature ${c}_j^2$ and the feature map *c*^2^:

3 $${c}_j^2=f\left({W}^2\otimes {c}_{j:j+k-1}^1+{B}^2\right)$$

4 $${c}^2=\left[{c}_1^2,{c}_2^2,{c}_3^2,\cdots, {c}_{N- 2}^2,{c}_{N- 1}^2,{c}_N^2\right]$$

where ⊗, *B* and *W* are the same as described above.

DeepACLSTM first applies the asymmetric convolution including two types of convolution operations to the representation matrix of proteins. Each type of convolutional operations have *M* filters. Thus the output of the convolution operation has *M* feature maps.

In order to generate the input of the stacked BLSTM neural networks, for each output of the second convolutional operation in the local context encoding module, we apply the fully connected (*FC*) layer with the ReLu activation function to get the input feature of BLSTM in protein sequences:

5 $$m={FC}^1\left({W}^m{c}^2+{B}^m\right)$$

Finally, the amino-acid sequence is represented as *m*: *m*_1_, *m*_2_, ⋯, *m*_*N* − 1_, *m*_*N*_.

In summary, CNNs [27] have the ability of capturing local relationships of spatial or temporal structures, but it only performs excellently in extracting n-gram features of amino acids at different positions of protein sequences through convolutional filters. In addition, long-distance interdependencies [3, 8, 24] of amino-acid residues are also critical for predicting PSS; therefore, the local complex features generated by asymmetric convolutions are fed into the stacked BLSTM to further extract long-distance interdependencies between amino-acid residues.

### Long-distance dependency encoding module

**Fig. 5:**
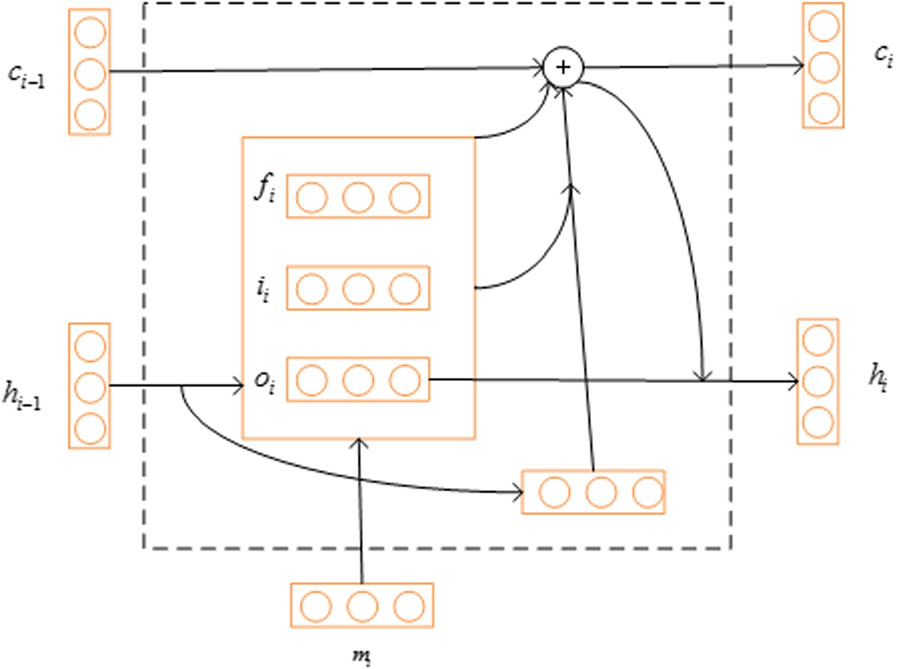
Internal architecture of the LSTM cell

The long-distance dependency encoding module includes two stacked BLSTM neural networks; this section describes the LSTM unit and explains how BLSTM neural networks can generate a fixed-length feature vector of each amino acid. Recurrent neural networks (RNNs) have achieved remarkable results in sequence modeling, but the gradient vector possible grows or degrades exponentially over long sequences during training [42]. Thus LSTM neural networks are designed to avoid the problems by introducing gate structures. LSTM [42, 43] neural networks are able to handle input sequences with arbitrary length via a transition function on a hidden vector *h*_*t*_, as the formula (10). Figure 5 represents the internal structure of a LSTM unit. At the time step *t*, the hidden vector *h*_*t*_ is computed by current input *m*_*t*_ received and its previous hidden vector *h*_*t* − 1_ at time *t*. LSTM utilizes three gates (input gate *i*_*t*_, forget gate *f*_*t*_ and output gate *o*_*t*_) and a memory cell *c*_*t*_ to control information processing of each amino acid at time step *t*.

Formally, the information of a LSTM unit can be computed by the following formulas:

6 $${f}_t= sigmoid\left({W}_f{m}_t+{W}_f{h}_{t-1}+{B}_f\right)$$

7 $${i}_t= sigmoid\left({W}_i{m}_t+{W}_i{h}_{t-1}+{B}_i\right)$$

8 $${c}_t={f}_t\otimes {c}_{t-1}+{i}_t\otimes \tanh \left({W}_c{m}_t+{W}_c{h}_{t-1}+{B}_c\right)$$

9 $${o}_t= sigmoid\left({W}_o{m}_t+{W}_o{h}_{t-1}+{B}_o\right)$$

10 $${h}_t={o}_t\otimes \tanh \left({c}_t\right)$$

Where *f*_*t*_, *i*_*t*_, *o*_*t*_ and *c*_*t*_ are the activation values of the forget gate, input gate, output gate and internal memory cell, respectively. Moreover, *W*, *B* and ⊗ respectively represent the weight matrix, bias term and element-wise multiplication.

**Fig. 6:**
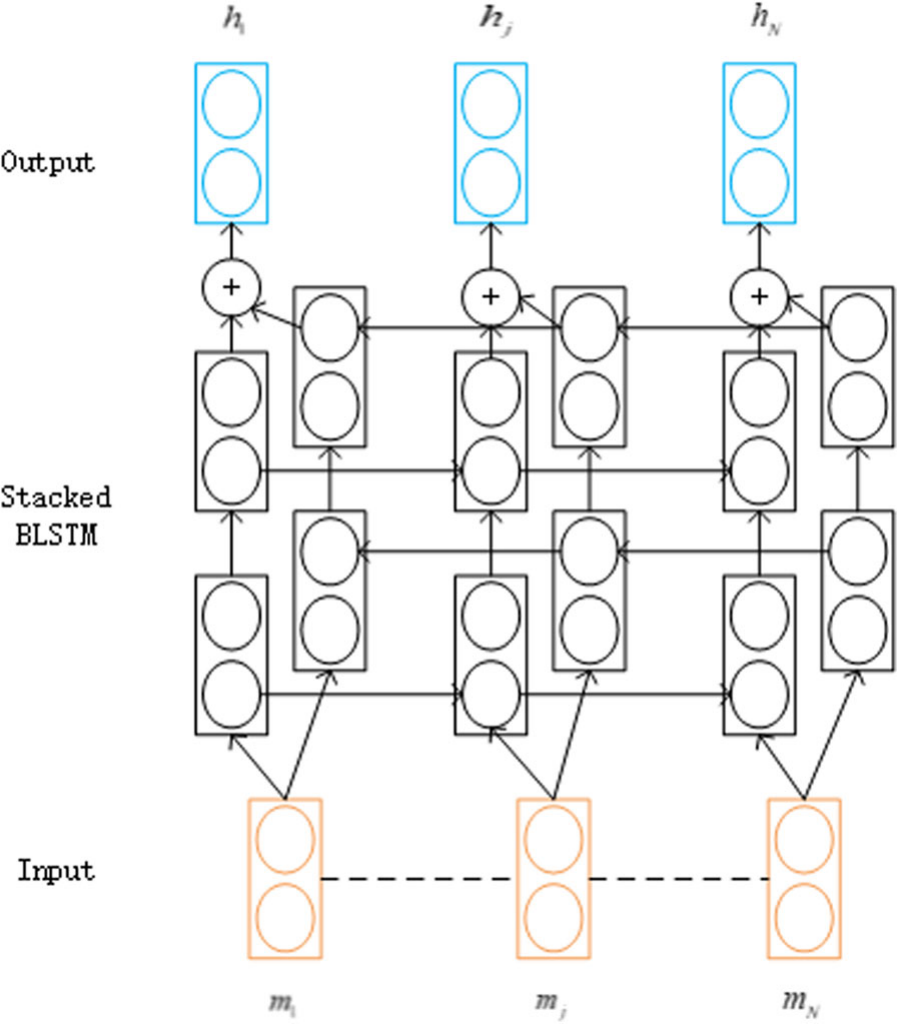
Architecture of stacked BLSTM neural networks

In our work, a BLSTM neural network consists of two LSTM neural networks in parallel, as showed in Fig. 6; one runs on the input sequence and the other runs on the reverse of the input sequence. We exploit two stacked BLSTM neural networks to capture more long-distance interdependencies of amino-acid residues. The first BLSTM neural network is exploited to protein sequences (*m*_1_, *m*_2_, ⋯, *m*_*N* − 1_, *m*_*N*_) at each time step to obtain a left-to-right sequence of hidden states $\overrightarrow{h^1}$ ($\overset{\rightharpoonup }{h_1^1},\overset{\rightharpoonup }{h_2^1},\cdots, \overset{\rightharpoonup }{h_{N-1}^1},\overset{\rightharpoonup }{h_N^1}$) and a right-to-left sequence of hidden states $\overleftarrow{h^1}$ ($\overleftarrow{h_1^1},\overleftarrow{h_2^1},\cdots, \overleftarrow{h_{N-1}^1},\overleftarrow{h_N^1}$); and then the second BLSTM neural network is exploited directly to obtain the same hidden states: $\overrightarrow{h^2}$ ($\overset{\rightharpoonup }{h_1^2},\overset{\rightharpoonup }{h_2^2},\cdots, \overset{\rightharpoonup }{h_{N-1}^2},\overset{\rightharpoonup }{h_N^2}$) and $\overleftarrow{h^2}$ ($\overleftarrow{h_1^2},\overleftarrow{h_2^2},\cdots, \overleftarrow{h_{N-1}^2},\overleftarrow{h_N^2}$) based on the previous hidden state vectors ($\overset{\rightharpoonup }{h_1^1},\overset{\rightharpoonup }{h_2^1},\cdots, \overset{\rightharpoonup }{h_{N-1}^1},\overset{\rightharpoonup }{h_N^1}$) and ($\overleftarrow{h_1^1},\overleftarrow{h_2^1},\cdots, \overleftarrow{h_{N-1}^1},\overleftarrow{h_N^1}$).

Finally, we concatenate the outputs of the second BLSTM neural network to obtain the final feature representation containing both the forward and backward information of each amino acid. The feature vectors of each residue at time step *t* by the second BLSTM neural network are:

11 $${h}_t= concat\left(\overrightarrow{h_t^2},\overleftarrow{h_t^2}\right)$$

### Prediction module

DeepACLSTM has two fully connected hidden layers in the prediction module. Moreover, in order to get the whole features of protein sequences, we concatenate local features *m* from the first fully connected layer and long-distance features $\overrightarrow{h^2},\overleftarrow{h^2}$ from long-distance dependency encoding module by the following formula:

12 $$h= concat\left(\overrightarrow{h^2},\overleftarrow{h^2},m\right)$$

The features of a protein sequence are finally recorded as $h=\left[{h}_1,{h}_1,{h}_1,\cdots, {h}_{N^{\prime }-2},{h}_{N^{\prime }-1},{h}_{N^{\prime }}\right]$, and then are fed into the first fully connected (*FC*) layer with the ReLU activation function to obtain the feature representation *h*^*f*^, by the following formula:

13 $${h}^f={FC}^2\left({\mathrm{W}}^hh+{B}^h\right)$$

Moreover, the feature representation *h*^*f*^ is fed into the second fully connected layer with the softmax activation function and performs 8-category secondary structure prediction by the formula:

14 $$y= softmax\left({\mathrm{W}}^s{h}^f+{B}^s\right)$$

The objective function of our method is to minimize the cross-entropy loss function.

## Data Availability

All data generated or analyzed during this work and the source codes for DeepACLSTM can be available online at https://github.com/GYBTA/DALSTM/.

## Abbreviations

ACLSTM: asymmetric convolutional long short-term memory
ACNNs: asymmetric convolutional neural networks
BLSTM: bidirectional long short-term memory
BRNNs: bidirectional recurrent neural networks
CNNs: convolutional neural networks
CRF: conditional random field
LSTM: Long short-term memory
PSS: protein secondary structure
PSSM: Position Specific Score Matrix
RNNs: recurrent neural networks
